# Mexico’s household health expenditure on diabetes and hypertension: What is the additional financial burden?

**DOI:** 10.1371/journal.pone.0201333

**Published:** 2018-07-27

**Authors:** Juan Pablo Gutierrez, Sebastian Garcia-Saiso, Belkis M. Aracena

**Affiliations:** 1 National Institute of Public Health, Cuernavaca, Morelos, Mexico; 2 Directorate of Quality and Health Education, Mexico City, Mexico; Public Library of Science, UNITED KINGDOM

## Abstract

**Objective:**

This study aimed to estimate the magnitude of the association between overall household health expenditures & the presence of members with a chronic disease in the household.

**Research design & methods:**

This was a cross-sectional analysis of a probabilistic household survey, which gathered data on previously diagnosed type 2 diabetes mellitus and hypertension as well as health expenditure in Mexico. From an analytic sample of 44,000 households, we identified those having at least one member with diabetes or hypertension and determined their health expenditure. Using matching procedures, we compared these data with those of households lacking such individuals.

**Results:**

We found that 24% of the households had at least one member who had been diagnosed with diabetes, hypertension, or both. Households with such members reported health expenditures that were 25%–34% (*P* <0.01) higher than households without such individuals. Such differences were more pronounced among households at lower socioeconomic levels and among those with no or limited health insurance.

**Conclusions:**

In addition to their impact on individual health, chronic ailments exert financial pressure on households. The additional health-care expenditure for households owing to such diseases leaves them financially exposed—especially households with lower income levels.

## Introduction

In 2012, non-communicable diseases (NCDs) accounted for 38 million deaths worldwide, representing 68% of all deaths; most of those deaths (28 million, 74%) occurred in low- or middle-income countries [1]. Among NCDs, the incidence of diabetes has increased rapidly in recent decades; it has become a global pandemic, affecting both developed and developing countries [2]. One estimate of the worldwide prevalence of diabetes suggested that in 2010 about 6.4% of individuals aged 20–79 years suffered from diabetes. That proportion is expected to increase to 7.7% by 2025 [3]. Hypertension is also a major health issue since it is an important risk factor for cardiovascular and kidney disease. According to one study, hypertension affected 26.4% of the global adult population in 2005, and it is expected to increase to 29.2% by 2025 [4].

One investigation for the United States determined that the total cost of diagnosed diabetes in 2012 was $245 billion; that figure for 2017, is expected to show a 41% increase [5]. That study found that individuals with diagnosed diabetes face medical expenditures 2.3 times higher than non-diabetics. Owing to both the prevalence of NCDs and high costs associated with their health consequences, their economic impact is considerable. For the period 2011–25, total economic losses due to those causes in low- and middle-income countries are estimated to be US$7 trillion [5]. Previous analyses have already highlighted the financial burden that NCDs impose on poor households in low- and middle-income countries; NCDs may in fact increase income inequalities [6]. This issue is particularly relevant with regard to health insurance coverage. People facing financial constraints may suffer an extra financial burden owing to their health, which could result in impoverishment [7].

In Mexico, the burden of chronic diseases has been extensively documented; the country is among those with the highest number of people living with NCDs [8, 9]. NCDs in Mexico account for over 47% of total premature deaths from all causes in men and for more than 67% in women. From a government perspective, designing interventions to address the high costs associated with these conditions has been a priority in recent years [9].

Based on the analysis of the disease burden, diabetes is the leading cause of years of healthy life lost in Mexico: the increased mortality from 1990 to 2010 was 82% [10]. Diabetes has become the main health problem in Mexico. The incidence of diagnosed diabetes among adults 20 years and older increased from 4.6% in 2000 (about 2.1 million individuals) to 9.2% in 2012 (6.4 million individuals): that is a 100% increase [11]. The disease is the second-leading cause of death: the increase in mortality from 1990 to 2010 was 82%. Based on analyses of disease burden, diabetes is the leading cause of years of healthy life lost [10]. Based on previous estimates of the diagnosed/undiagnosed ratio, a total prevalence of at least 15% was expected in 2012[12, 13]. The Organisation for Economic Co-operation and Development estimates of diabetes in Mexico suggest a total prevalence of 15.9%—the highest among member countries [14]. The financial burden of the disease will increase owing to its early onset: 23.8% of people aged 40–59 years and 5.9% for those aged 20–39 years. This will result in extended utilization of health services and productivity loss. The same applies to hypertension, especially for young people with cardiovascular diseases [1].

The prevalence of hypertension, one of the most important risk factors for cardiovascular disease [4], remained relatively stable between 2000 and 2012 in Mexico: it increased from 30.1% to 31.4% among adults aged 20 years and older [11]. However, the number of people unaware of their high blood pressure could be problematic. In 2012, 47.3% of individuals diagnosed with hypertension were previously unaware of their blood pressure status [15].

An analysis of individuals diagnosed with diabetes in Mexico revealed that about half of them were also diagnosed with hypertension [12]. The distribution of diabetes and hypertension across the country shows marked heterogeneity among states: variations range from 4.8% among males in Chiapas to 15.5% among women in Nuevo León [12].

Having to deal with the treatment and care of NCDs may constitute a major financial burden for affected households: it could push them into poverty, especially those without public health coverage [16]. However, the cost for people living with diabetes or hypertension is unknown—even though estimating the financial burden of those suffering from such ailments is relevant in terms of pursuing universal health coverage and ensuring effective access to health services. According to one estimate, the cost of managing diabetes in Mexico varies considerably by institution: from $700 to $3,200 a year [12]. This discrepancy underlines the need for obtaining precise estimates based on household data.

The objective of the present study was to determine the differences in health expenditure among households with a member diagnosed as having diabetes or hypertension; we then aimed to compare those results with the health expenditure of households without such individuals. That would allow us to estimate the magnitude of the financial burden those conditions imposed on Mexican households. In addition, we assessed if differences in that burden were related to the type of health insurance.

## Research design and methods

In this paper, we report a cross-sectional analysis of data from a national probabilistic survey, the National Health and Nutrition Survey 2012 (ENSANUT 2012). A detailed description of the survey design and sampling is reported elsewhere [17]. Briefly, the ENSANUT 2012 was a national- and state-level (subnational) representative survey of the Mexican population conducted in 2012; the survey was stratified for rural and urban populations.

The ENSANUT 2012 collected data from 50,238 households. The survey obtained information on general dwelling characteristics as well as household and individual characteristics. Details were provided by an adequate household informant, who was defined as a current resident of legal age with sufficient knowledge of all household members. Data were also obtained from one randomly selected individual in each of the following age-ranges at each household: up to 9 years; 10–19 years; and 20 years or older (regarded as adults). The analytic sample comprised 44,000 households (with data from at least one adult). Among a set of health conditions, selected adults were asked about existing diagnoses of diabetes and hypertension. That information was used to identify households having at least one member with diabetes, hypertension, or both.

We used data reported at the household level to determine total expenditure per household, health expenditure per household, and (based on those two variables) health expenditure as a percentage of total household expenditure. The survey covered household health expenditure for the last quarter (last 3 months; see the specific wording of the items in the questionnaire in the S1 Table). It included the following categories: inpatient services; outpatient services; drugs; laboratory and other diagnostic services; and additional health services. Expenditure data were obtained from a household key informant (usual resident of the household of 18 years or older), who may or may not have been the same person who provided information about health conditions (because of random sampling of individuals form household members´ roster, the household informant may or may not being sampled). Expenditure data were collected for the household, not for specific individuals; thus, it was not possible to distinguish between individual health-related expenditure and household health expenditure.

Details of financial protection for health were derived from the health insurance status of the reporting adult. We categorized that status as uninsured, social security, or Seguro Popular (health insurance for individuals without social security).

In the present study, we also used the socioeconomic indicator generated for ENSANUT 2012. Details on this indicator are published elsewhere; briefly, this indicator categorized households into socioeconomic quintiles and was constructed by estimating household income levels [18].

We report descriptive statistics comparing households with and without members in the previously described categories (diabetes, hypertension, or both) with their confidence intervals.

We recognized the potential selection bias related to the characteristics of households with individuals having either or both of the described diagnoses; thus, we implemented ex-post matching based on characteristics that could be assumed to be independent of time. Using matching estimations allows a comparison of households that are similar in most characteristics except that of having members with chronic conditions [19–21]. We estimated differences in health expenditure and proportion of total health-related expenditure using an average treatment effect approach.

Estimation models adopted 1:1 propensity-score matching (PSM) and nearest-neighbor matching (NNM). With the two approaches, matching variables were as follows: size of household (number of members); number of school years for head of household; number of school years for spouse of head of household; sex and age of head of household; number of dependents; marginalization index at locality level; state of residence; and household quintile. Ordinarily, household size affects general expenditure: larger households have greater expenditure. Schooling is related to both overall socioeconomic status and health knowledge; thus, it is related to health expenditure. Locality variables reflect socioeconomic conditions and availability of health services. The NNM identifies the closest observation based on a set of defined covariates using the Mahalanobis distance. PSM is a technique that uses an index of the characteristics of the analytic units. In the present study, those characteristics involved the conditional probability of a household member having a chronic condition. With that index, the closer the proximity of two observations, the greater is the likelihood that they have sufficient similarity to allow comparison [22]. As noted above, we selected those variables because of their relatively time-invariant characteristics so as to reflect pre-disease conditions. PSM has previously been used to determine health expenditure in Mexico [23, 24].

We undertook our analyses using Stata, version 13.1 (StataCorp, College Station, TX, USA).

Data collection and analysis of ENSANUT 2012 were approved by the National Institute of Public Health in Mexico Committee of Ethics on Research, which meets both Mexican and US requirements for human subject protection. Informed consent was obtained from all participants following procedures reviewed and approved by that ethics committee.

## Results

Among all the analyzed households, 11% (n = 4,403) had at least one member who had been diagnosed with diabetes; 18% had at least one member diagnosed with hypertension (n = 7,606); and 24% (n = 9,900) had at least one member diagnosed with either diabetes or hypertension. On average, the quarterly household health expenditure was 884 pesos (about US$44.2). When comparing households having members diagnosed with either diabetes or hypertension with those lacking such individuals (Table 1), quarterly household health expenditure was greater among the former than the latter.

**Table 1:** Average (95% confidence intervals) household quarterly health expenditures (in pesos) among Mexican households according to presence of household members with diagnosed diabetes or hypertension.

	Households having at least one member with diabetes		Households having at least one member with hypertension		Households having at least one member with diabetes or hypertension	
	No	Yes	No	Yes	No	Yes
**All**	855.46	1,127.45	806.96	1,228.05	795.77	1,169.15
	(808.94–901.98)	(985.59–1,269.30)	(763.69–850.23)	(1,092.31–1,363.79)	(751.06–840.48)	(1,057.12–1,281.17)
**1st quintile**	225.87	264.90	215.45	311.40	215.00	289.91
	(204.89–246.85)	(178.53–351.28)	(197.05–233.86)	(230.82–391.99)	(196.02–233.98)	(225.81–354.00)
**2nd quintile**	415.95	492.65	403.37	522.70	402.28	497.54
	(380.23–451.66)	(377.93–607.37)	(370.34–436.40)	(405.67–639.73)	(367.32–437.25)	(405.39–589.70)
**3rd quintile**	583.02	644.91	557.90	717.82	558.75	681.66
	(535.66–630.38)	(511.60–778.21)	(512.05–603.75)	(598.46–837.18)	(511.79–605.71)	(580.12–783.20)
**4th quintile**	975.10	1,182.61	942.07	1,223.83	934.38	1,184.57
	(894.99–1,055.22)	(986.99–1,378.22)	(863.71–1,020.43)	(1,033.04–1,414.63)	(851.65–1,017.10)	(1,020.87–1,348.26)
**5th quintile**	2,193.57	2,839.20	2,062.66	3,075.31	2,029.87	2,945.21
	(2,006.40–2,380.75)	(2,267.81–3,410.59)	(1,887.43–2,237.89)	(2,513.59–3,637.02)	(1,851.07–2,208.67)	(2,482.65–3,407.77)

**Fig 1:**
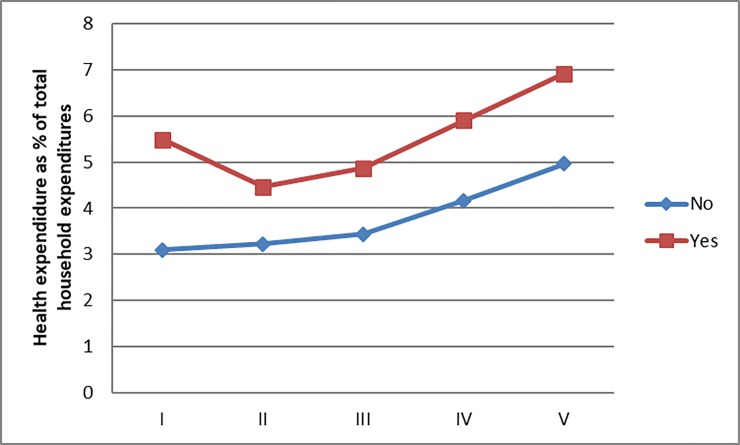
Mexican household health expenditure as proportion of total expenditure according to presence of a member with diagnosed diabetes or hypertension.

Those differences remained even after stratifying by socioeconomic quintiles. For each quintile, quarterly household health expenditure was greater among households having members with the chronic diseases than households lacking such individuals. Table 2 presents the proportion of total household expenditure devoted to health care. As evident in that table, household health expenditure as a percentage of total expenditure was greater among households having members diagnosed with diabetes or hypertension than among households without such individuals. The differences were as follows: 39% higher in households having members with diabetes; 52% higher in households having members with hypertension; and 49% higher in households having members with both conditions. As indicated in Fig 1, those differences were greater for the first quintile and statistically significant (*P* <0.01). This finding suggests that among households with higher economic constraints, the additional financial burden of those conditions was even greater.

**Table 2:** Average (95% confidence interval) percentage of total household health among Mexican households according to presence of a member with diagnosed diabetes or hypertension.

	Households having at least one member with diabetes		Households having at least one member with hypertension		Households having at least one member with diabetes or diabetes	
	No	Yes	No	Yes	No	Yes
**All**	4.02	5.57	3.82	5.79	3.75	5.58
	(3.87–4.17)	(5.09–6.05)	(3.68–3.97)	(5.40–6.19)	(3.61–3.89)	(5.22–5.95)
**1st quintile**	3.46	4.53	3.17	5.87	3.10	5.49
	(3.13–3.79)	(3.32–5.74)	(2.86–3.47)	(4.71–7.03)	(2.82–3.38)	(4.33–6.65)
**2nd quintile**	3.35	4.85	3.31	4.47	3.23	4.46
	(3.12–3.58)	(3.81–5.88)	(3.07–3.54)	(3.79–5.14)	(2.99–3.47)	(3.86–5.06)
**3rd quintile**	3.68	4.79	3.47	5.09	3.44	4.87
	(3.43–3.93)	(3.88–5.69)	(3.22–3.73)	(4.43–5.76)	(3.17–3.70)	(4.29–5.44)
**4th quintile**	4.42	6.04	4.22	6.14	4.16	5.91
	(4.14–4.70)	(5.17–6.92)	(3.96–4.48)	(5.33–6.94)	(3.88–4.43)	(5.20–6.62)
**5th quintile**	5.25	7.28	5.07	7.10	4.97	6.92
	(4.91–5.58)	(6.12–8.43)	(4.71–5.42)	(6.30–7.90)	(4.61–5.33)	(6.23–7.61)

**Fig 2:**
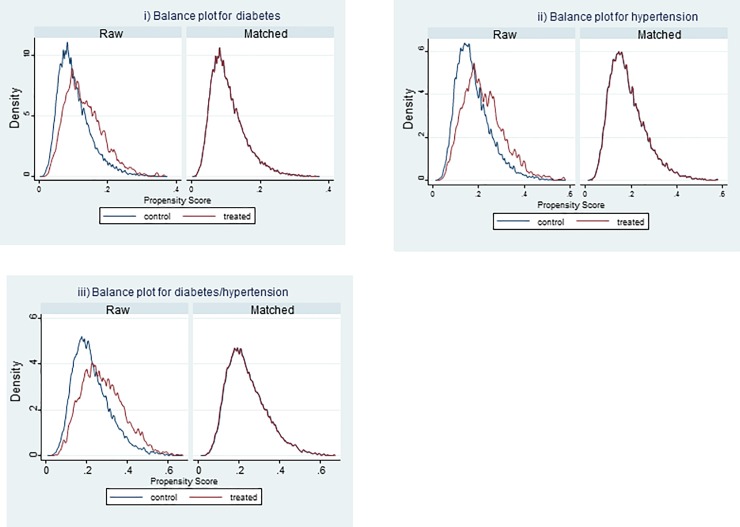
Balance plots for the matching procedure: i) households having members with diabetes; ii) households having members with hypertension; iii) households having members with either diabetes or hypertension.

Fig 2 presents balance plots for the matching. As evident in that figure, matching facilitated comparisons among the groups: it resulted in groups that were similar in terms of time-invariant characteristics related to expenditure. When evaluating the differences among the groups using matching, the differences in health expenditure and as a proportion of total health expenditure were greater than 0 in all cases. As indicated in Table 3, household expenditure was 162–284 pesos (about US$7.3–15.8; i.e., 19%–27%) higher in households having at least one member with diabetes than those without such individuals. The differences were greater for households lacking health insurance. With respect to type of insurance, the differences were greater for households having Seguro Popular than those with social security.

**Table 3:** Difference (95% confidence interval) in total expenditure and percentage of total household health among Mexican households having a member with diagnosed diabetes or hypertension matched with households without such individuals.

	Health expenditure		Health expenditures as % of total expenditure	
All households	PSM	NNM	PSM	NNM
**Households having members with diabetes**	233.02***	162.48***	0.59***	0.62***
	(231.13–234.90)	(161.24–163.72)	(0.59–0.60)	(0.61–0.62)
**Households having members with hypertension**	233.02***	285.32***	0.99***	1.02***
	(230.89–235.16)	(284.00–286.64)	(0.99–1.00)	(1.01–1.02)
**Households having members with diabetes or hypertension**	273.24***	220.53***	0.97***	0.82***
	(271.49–274.99)	(219.27–221.78)	(0.96–0.98)	(0.81–0.82)
**Uninsured households**	PSM	NNM	PSM	NNM
**Households having members with diabetes**	400.46***	707.67***	1.98***	2.73***
	(396.46–404.46)	(704.75–710.59)	(1.96–1.99)	(2.72–2.74)
**Households having members with hypertension**	372.52***	571.43***	1.91***	2.20***
	(368.90–376.15)	(568.59–574.27)	(1.90–1.93)	(2.19–2.21)
**Households having members with diabetes or hypertension**	323.76***	569.93***	1.60***	2.06***
	(320.28–327.25)	(567.11–572.75)	(1.59–1.62)	(2.05–2.07)
**Seguro Popular households**	PSM	NNM	PSM	NNM
**Households having members with diabetes**	148.09***	121.37***	0.38***	0.26***
	(144.74–151.43)	(118.92–123.83)	(0.38–0.39)	(0.25–0.27)
**Households having members with hypertension**	470.71***	463.16***	0.80***	1.10***
	(467.02–474.41)	(460.66–465.66)	(0.79–0.81)	(1.09–1.11)
**Households having members with diabetes or hypertension**	330.51***	387.26***	0.72***	0.93***
	(327.00–334.01)	(384.79–389.74)	(0.71–0.73)	(0.93–0.94)
**Social security households**	PSM	NNM	PSM	NNM
**Households having members with diabetes**	57.40***	123.10***	1.01***	0.78***
	(55.36–59.44)	(121.62–124.59)	(1.00–1.02)	(0.77–0.78)
**Households having members with hypertension**	102.16***	171.66***	0.59***	0.95***
	(100.09–104.23)	(170.17–173.14)	(0.58–0.59)	(0.94–0.96)
**Households having members with diabetes or hypertension**	103.04***	93.94***	0.61***	0.66***
	(100.86–105.21)	(92.47–95.41)	(0.60–0.62)	(0.65–0.66)

PSM, propensity score matching; NNM, nearest neighbor match

*** p<0.01

** p<0.05

* p<0.1

For households having members with hypertension, health expenditure was 233–285 pesos (about $11.1–15.4; i.e., 29%–35%) higher than those without such diagnosis. The differences in terms of type of insurance were similar to those for diabetes: households lacking insurance had an additional burden of 463–470 pesos. For households having at least one member with either diabetes or hypertension, health expenditure was 220–273 pesos (about $10.5–13.0; i.e., 28%–34%) higher than those without such individuals.

With respect to proportion of total health expenditure, households having members with diabetes had 0.59–0.62 percentage points higher expenditure (i.e., 15%) than households without such individuals. For hypertension, the greater expenditure amounted to 0.99–1.02 percentage points (i.e., 26%–27%). For either of the two conditions, the greater expenditure was 0.82–0.97 percentage points (i.e., 22%–26%).

## Discussion

This paper offers a quantitative estimate of the additional financial burden to which Mexican households are exposed with respect to chronic diseases, particularly diabetes and hypertension. We found that health expenditure as a proportion of total household expenditure increased on average by 25% among households having members diagnosed with one of those conditions. That difference was up to 77% among households in the lowest income quintile: 5.49% versus 3.10% [25].

Households in the lowest income quintile having members with diabetes or hypertension reported a higher financial burden, and Seguro Popular covered a large proportion of those households: this represents an important challenge for that insurance scheme and its public providers. We found that households having members with either diabetes or hypertension under Seguro Popular insurance faced a heavier financial burden than those under social security insurance. This may result in differential access to medicines, exacerbating the situation for an already financially vulnerable population.

The relationship between household expenditure and chronic disease has been previously examined. In particular, the challenge for low- and middle-income countries has been highlighted [6]; however, not many studies have addressed this issue [26]. In Nepal, Saito et al. reported a positive correlation between hypertension and household expenditure [27]. One investigation in Mexico reported the financial burden for households having members with diabetes: it found a high proportion of out-of-pocket expenditures for health care there [25]. In India, Ramachandran et al, determined that diabetics spent up to 30% of the household income on health care [28]. For Mexico, one recent analysis used the same survey data as the present study (ENSANUT 2012); however, it estimated costs using unit costs from other sources rather than directly reported expenditure, as examined here. That earlier study found that chronic diseases may account for up to 25% of total expenditure among the main care providers in Mexico [29]. Estimations based on modeling in Mexico suggest that diabetes-related expenditure can have catastrophic consequences [30].

The increased financial burden associated with diabetes and hypertension may also constitute a barrier for seeking health care. It has been reported that a major proportion of ambulatory care is provided in the private sector through out-of-pocket expenditure [31]; this poses a threat to the provision of continuous care. The extra financial burden for health care among households may be exacerbated by drug shortages at public facilities. Data from the ENSANUT 2012 survey indicate that only 66%–86% of users of public health services received their prescribed medicines at the public facilities they attended [11].

One study found that access to health services in Mexico was related to health insurance and also to other social determinants of health: being poor and living in small localities decreased the probability of using ambulatory health services [32]. To tackle this additional financial burden, it is necessary to address both health insurance and the structural quality of health services; with the latter, that particularly applies to the provision of all prescribed drugs [33].

One important limitation of the present study is that ENSANUT was not designed to obtain detailed information about household expenditure. As with all self-reported data, reported expenditure may be affected by recall bias; however, that should affect all households. In a comparison of households with the same survey data, that limitation should equally affect households both having and lacking members diagnosed with chronic diseases. Thus, we do not believe that this limitation affected the results. It would be desirable for similar estimates to consider household income; however, that would not be possible using the ENSANUT data. In addition, not all adult residents were interviewed in the survey; thus, there may have been unreported household members with diabetes or hypertension. The sample in the present study was probabilistic: we would expect such other unreported household members to be distributed among all household types, thereby cancelling out the potential bias. Diabetes and hypertension are related to other health conditions (e.g., obesity, heart disease); accordingly, the additional financial burden may reflect those other conditions and not be limited to diabetes and hypertension.

As documented in other studies [30], chronic diseases can have negative impacts on individuals in addition to their health effects; that additional impact can lead to a deterioration in household living conditions. The financial burden may result in reduced access to health care, resulting in further health deterioration. Increased health-care expenditure may represent an opportunity cost with respect to other household expenditures. This problem is compounded when considering the presumed reduction in income owing to health problems, and it constitutes an important impoverishment factor.

## Supporting information

S1 Table Specific wording of questions on health expenditures from ENSANUT 2012. (DOCX)

## References

[1] WHO. Global status report on noncommunicable diseases 2014. Geneva: World Health Organization; 2014.

[2] van Dieren S, Beulens JW, van der Schouw YT, Grobbee DE, Neal B. The global burden of diabetes and its complications: an emerging pandemic. Eur J Cardiovasc Prev Rehabil. 2010;17(1 suppl): s3–s8. 10.1097/01.hjr.0000368191.86614.5a 20489418

[3] Shaw JE, Sicree RA, Zimmet PZ. Global estimates of the prevalence of diabetes for 2010 and 2030. Diabetes Research and Clinical Practice. 2010;87(1): 4–14. 10.1016/j.diabres.2009.10.007 19896746

[4] Kearney PM, Whelton M, Reynolds K, Muntner P, Whelton PK, He J. Global burden of hypertension: analysis of worldwide data. The Lancet. 2005;365(9455): 217–223. 10.1016/S0140-6736(05)17741-1 15652604

[5] ADA. Economic costs of diabetes in the U.S. in 2012. Diabetes Care. 2013;36(4):1033–46. 10.2337/dc12-2625 23468086 PMC3609540

[6] Kankeu HT, Saksena P, Xu K, Evans DB. The financial burden from non-communicable diseases in low- and middle-income countries: a literature review. Health Res Policy Syst. 2013;11(1): 31 10.1186/1478-4505-11-31 23947294 PMC3751656

[7] Goryakin Y, Suhrcke M. The prevalence and determinants of catastrophic health expenditures attributable to non-communicable diseases in low- and middle-income countries: a methodological commentary. International Journal for Equity in Health. 2014;13(1): 107 10.1186/s12939-014-0107-1 25376485 PMC4228103

[8] FID. Atlas de Diabetes: Federación Internacional de Diabetes; 2012. Available from: http://www.idf.org/diabetesatlas/5e/Update2012. Cited December 2012.

[9] Córdova Villalobos JA, Barriguete Meléndez JA, Lara Esqueda A, Barquera S, Rosas Peralta M, Hernández Ávila M. Las enfermedades crónicas no transmisibles en México: sinopsis epidemiológica y prevención integral. Salud Publica Mex. 2008;50: 419–427. 18852939 10.1590/s0036-36342008000500015

[10] Lozano R, Gómez-Dantés H, Pelcastre B, Ruelas G, Montañez JC, Capuzano C, et al Carga de la enfermedad en México, 1990–2010. Nuevos resultados y desafíos Cuernavaca, Mexico: Instituto Nacional de Salud Pública / Secretaría de Salud; 2014.

[11] Gutiérrez JP, Rivera-Dommarco J, Shamah-Levy T, Villalpando-Hernández S, Franco A, Cuevas-Nasu L, et al Encuesta Nacional de Salud y Nutrición 2012 Resultados Nacionales. Cuernavaca, Mexico: Instituto Nacional de Salud Pública (MX); 2012.

[12] Hernández-Ávila M, Gutiérrez JP, Reynoso-Noverón N. Diabetes mellitus en México: El estado de la epidemia. Salud Pública de México. 2013;55: s129–s136. 24626688

[13] Villalpando S, Shamah-Levy T, Rojas R, Aguilar-Salinas CA. Trends for type 2 diabetes and other cardiovascular risk factors in Mexico from 1993–2006. Salud Publica Mex. 2010;52: S72–S79. 20585732 10.1590/s0036-36342010000700011

[14] OECD (2016), OECD Reviews of Health Systems: Mexico 2016, OECD Publishing, Paris 10.1787/9789264230491-en

[15] Campos-Nonato I, Hernández-Barrera L, Rojas-Martínez R, Pedroza A, Medina-García C, Barquera-Cenera S. Hipertensión arterial: prevalencia, diagnóstico oportuno, control y tendencias en adultos mexicanos. Salud Publica Mex. 2013;55: S144–S150. 24626690

[16] Arredondo A. Type 2 diabetes and health care costs in Latin America: exploring the need for greater preventive medicine. BMC Medicine. 2014;12: 136 10.1186/s12916-014-0136-z 25266304 PMC4243717

[17] Romero-Martínez M, Shamah-Levy T, Franco-Núñez A, Villalpando S, Cuevas-Nasu L, Gutiérrez JP, et al Encuesta Nacional de Salud y Nutrición 2012: diseño y cobertura. Salud Publica Mex. 2012;55(S2): 332–340. 24626712

[18] Gutiérrez JP. Clasificación socioeconómica de los hogares en la ENSANUT 2012. Salud Publica Mex. 2013;55: S341–S346. 24626713

[19] Rosenbaum PR, Rubin DB. The central role of the propensity score in observational studies for causal effects. Biometrika. 1983;70(1): 41–55. PubMed PMID: ISI:A1983QH66900005.

[20] Heckman J, Ichimura H, Smith J, Todd P. Characterizing selection bias using experimental data. Econometrica. 1998;66(5): 1017–1098. PubMed PMID: ISI:000075653500001.

[21] Heckman JJ, Ichimura H, Todd PE. Matching as an econometric evaluation estimator: Evidence from evaluating a job training programme. Review of Economic Studies. 1997;64(4): 605–654. PubMed PMID: ISI:A1997YF75800006.

[22] Ravallion M. The mystery of the vanishing benefits: Ms Speedy Analyst’s introduction to evaluation. The World Bank Economic Review. 2001;15(1): 115–140.

[23] Ávila-Burgos L, Serván-Mori E, Wirtz VJ, Sosa-Rubí SG, Salinas-Rodríguez A. Efectos del Seguro Popular sobre el gasto en salud en hogares mexicanos a diez años de su implementación. Salud Publica Mex. 2013;55: S91–S99. 24626719

[24] Wirtz VJ, Santa-Ana-Tellez Y, Servan-Mori E, Avila-Burgos L. Heterogeneous effects of health insurance on out-of-pocket expenditure on medicines in Mexico. Value in Health. 2012;15(5): 593–603. 10.1016/j.jval.2012.01.006 22867767

[25] Arredondo A, Barceló A. The economic burden of out-of-pocket medical expenditures for patients seeking diabetes care in Mexico. Diabetologia. 2007;50(11): 2408–2409. 10.1007/s00125-007-0828-4 17879080

[26] Muka T, Imo D, Jaspers L, Colpani V, Chaker L, van der Lee SJ, et al The global impact of non-communicable diseases on healthcare spending and national income: a systematic review. Eur J Epidemiol. 2015;30(4): 251–277. 10.1007/s10654-014-9984-2 25595318

[27] Saito E, Gilmour S, Rahman MM, Gautam GS, Shrestha PK, Shibuya K. Catastrophic household expenditure on health in Nepal: a crosssectional survey. Bull World Health Organ. 2014;92: 760–767. 10.2471/BLT.13.126615 25378730 PMC4208475

[28] Ramachandran A, Ramachandran S, Snehalatha C, Augustine C, Murugesan N, Viswanathan V, et al Increasing expenditure on health care incurred by diabetic subjects in a developing country: a study from India. Diabetes Care. 2007;30(2): 252–256. 10.2337/dc06-0144 17259490

[29] Figueroa-Lara A, Gonzalez-Block MA, Alarcon-Irigoyen J. Medical expenditure for chronic diseases in Mexico: the case of selected diagnoses treated by the largest care providers. PLoS ONE. 2016;11(1): e0145177 10.1371/journal.pone.0145177 26744844 PMC4706295

[30] Arredondo A, Reyes G. Health disparities from economic burden of diabetes in middle-income countries: evidence from México. PLoS ONE. 2013;8(7): e68443 10.1371/journal.pone.0068443 23874629 PMC3709919

[31] Gutierrez JP, Garcia-Saiso S, Fajardo-Dolci G, Hernandez-Avila M. Effective access to health care in Mexico. BMC Health Services Research. 2014;14(1): 186 10.1186/1472-6963-14-186 24758691 PMC4006670

[32] Bautista-Arredondo S, Serván-Mori E, Colchero MA, Ramírez-Rodríguez B, Sosa-Rubí SG. Análisis del uso de servicios ambulatorios curativos en el contexto de la reforma para la protección universal en salud en México. Salud Publica Mex. 2014;56: 18–31. 24912517

[33] García-Saisó S, Gutiérrez JP, Pacheco-Estrello P, et al Los retos de la calidad de los servicios de salud como barreras para el acceso efectivo In: García-Saisó S, Hernández-Torres F, editors. La calidad de la atención a la salud en México a través de sus instituciones. Biblioteca Mexicana del Conocimiento. 2d ed. México, D.F.: Secretaría de Salud; 2015.

