# Supplementary material for: Mexico’s household health expenditure on diabetes and hypertension: What is the additional financial burden?
Source: PLoS One. 2018 Jul 27;13(7):e0201333. doi: 10.1371/journal.pone.0201333 (PMC6063432; doi:10.1371/journal.pone.0201333)
Supplement: S1 Table — (DOCX) [file pone.0201333.s001.docx]

Appendix: Questions on health expenditures

Table S1. Specific wording of questions on health expenditures from ENSANUT 2012

In the last 3 months, how much was spend by household members in…

…care for staying overnight at a hospital or health facility?

…care or physician/nurse/health provider services that didn´t require overnight stay, not including medicines?

… care by traditional heath providers (healers) including traditional treatments?

…dentist?

…medicines, excluding traditional treatments?

…glasses and prosthesis?

…diagnosis and laboratory tests, including x-ray and blood tests?

…any other health service not mentioned?
